# Globalization of Behavioral Risks Needs Faster Diffusion of Interventions

**Published:** 2007-03-15

**Authors:** Shahul Ebrahim, Joxel Garcia, Achmed Sujudi, Hani Atrash

**Affiliations:** National Center on Birth Defects and Developmental Disabilities; Pan American Health Organization, Washington, DC; Ministry of Health, Indonesia; National Center on Birth Defects and Developmental Disabilities, Centers for Disease Control and Prevention, Atlanta, GA

## Abstract

International trade, population migration, changes in living conditions (i.e., consumption transition, nutritional transition), and changes in production, marketing, and availability of consumer goods (i.e., production transition) have brought about continuous and rapid changes in the human environment. Such changes have improved the health and economic status of many people in developing countries. At the same time, a parallel phenomenon is occurring: the rapid emergence and expansion of modifiable risk behaviors. These behaviors adversely affect the national health of developing countries and that of future generations because of their impact on maternal, child, and adolescent health. Furthermore, these behaviors are increasing at a faster rate than interventions to curb their growth are being implemented. We discuss the current status of five modifiable risk behaviors — alcohol consumption, tobacco use, overweight and obesity, low fruit and vegetable consumption, and physical inactivity — to emphasize the need for global advocacy and local action to enhance policy formulation and diffusion of interventions necessary to moderate the spread of these behaviors.

## Introduction

More than three decades have passed since an editorial from the Second World Conference on Smoking and Health warned, "There is a real danger in this deadly habit [smoking] being exported to the younger countries of Africa and Asia, and the rest of the world has a responsibility to see that this is not done" (1). Although the expansion of the tobacco epidemic to the developing world continues, international public health has since benefited from an unprecedented global and legislative effort to curb it. At the same time, public health threats of equal or greater magnitude have emerged onto the global disease landscape. Many of these diseases result from modifiable risk behaviors.

Modifiable risk behaviors, some of which have traditionally been considered problems of the Western world or the cause of diseases of affluence (e.g., obesity, type 2 diabetes melitus), now rank from the first to 15th leading causes of health burden in developing countries (Table) (2). To illustrate their impact on public health in the developing world, we examine five of these behaviors: alcohol consumption, tobacco use, overweight and obesity, physical inactivity, and low fruit and vegetable intake. Some of these behaviors, such as overweight and obesity, are often classified as adverse outcomes of long-term exposure to other factors; however, in this article we approach all five as risk behaviors. Also, some reports have argued that children acquire behavioral risks in the same manner as they acquire communicable diseases (3).

Globalization, by definition, is the act, process, or policy of making something worldwide in scope or application. International interactions that arise from globalization, such as trade, migration, and increased access to information and marketing of goods, continuously modify the human environment. In many respects, the effects of globalization have been positive. Economic globalization appears to have raised the gross domestic product of developing countries and the standard of living of developing countries' poorer citizens. Such changes have led to improvement in some health indicators, for example, life expectancy, which was increasing in all countries prior to the onset of the AIDS epidemic (4). Globalization of technology and information is also expected to mitigate the inequity observed between the diffusion of public health interventions to the rich and to the poor (i.e., the inverse equity hypothesis) (5). However, not all outcomes of globalization have been favorable. The role of global changes and their sequelae in facilitating emergence or cross-border transfer of microbial disease has raised concern in recent years about how globalization may adversely affect other aspects of public health (4). The health implications of long-term changes in population behaviors have received less attention in affected countries than has the short-term threat that microbial disease transmission poses (6,7). Because advocacy is an important part of national health planning and given the evolving life-course approach to disease prevention and health promotion, it is important that all countries consider the public health threat that modifiable risk behaviors pose.

## Burden and Challenges

The growing health risks  associated with economic globalization in developing countries are not byproducts of globalization but, rather, the result of direct efforts (4). Alcohol and tobacco provide striking examples. Use of these two products has declined over the past two decades in Western countries; however, in developing countries where these behaviors are associated with social status and are considered desirable, use has increased (2,8). Although data for time trend analysis are not readily available for all regions, in the World Health Organization's (WHO's) designated 14 subregions of the world, alcohol consumption rates among adults range from 5% to 84%, and smoking rates range from 31% to 61% among men and 3% to 24% among women (2). Such high rates may have resulted from the efforts of international alcohol and tobacco companies, which have employed strong marketing techniques to penetrate new markets in the developing world, making their products household names. Traditional, locally produced alcoholic beverages and tobacco products, the use of which was limited to a small population subgroup comprising mostly men, were not subjected to similar high-intensity marketing strategies. Consequently, these local products are being supplanted by more potent and easily accessible industrially produced ones (i.e., production transition). In spite of their negative health impact, such changes are sometimes perceived as a measure of development. Furthermore, the accessibility of tobacco and alcohol products can create an added burden on poor communities because the elasticity of the market for these products may shift consumer priorities, creating a preference for alcohol and tobacco over more nutritious food items.

Changes in consumption patterns are not limited to alcohol and tobacco products but extend to recreation and communication. Globalization has brought about the development of new industries and technologies and their associated suppliers and support systems, which have replaced many of the historically rural and agricultural economies around the globe. These changes have resulted in built environments, that is, construction of industrial complexes and communities and their associated living centers close to existing cities or to new planned and unplanned urban centers. It is estimated that by 2007, more than 50% of the world's population will be living in urban areas (2). The environment in these urban complexes is associated with a more sedentary lifestyle than that of traditional agricultural societies. The increased use of television and other communication technologies for learning and recreation has created new sedentary pastimes, especially among children, seniors, and the poor. Among adults in WHO's 14 subregions, physical inactivity rates range from 11% to 24% (2).

Physical inactivity, combined with the increased availability, accessibility, and consumption of manufactured foods high in fat and sugars, has led to overnutrition (i.e., nutritional transition) and an epidemic of overweight and obesity (9). Obesity now challenges undernourishment, the historic, nutrition-associated global public health threat. The presence of both underweight and overweight among the top 10 causes of health burden (fourth and fifth leading causes of DALYs [disability-adjusted life years] in low-mortality developing countries) reflects a paradox: underweight represents a development failure, and overweight represents a development success (2). This paradox is more pronounced among populations that migrate, either within a country or between countries.

For some people, whether because of the role of the "thrifty gene" (10) or because of eating behavior, undernutrition may have given way to obesity. In October 2004, the Chinese health ministry reported that between 1992 and 2002 obesity in China doubled (11). That estimate showed that in 2002, 7.1% of the Chinese population (60 million people) were obese, and 22.8% (200 million) were overweight, edging China past the United States in its number of overweight people (11). In 2001, one third of Costa Rican children were found to be overweight, and obesity rates in excess of 15% have been reported for several countries of Latin America. Overweight now exceeds underweight among women in 36 developing countries (12,13).

Developed countries have had a slower transition to epidemics of modifiable risk behaviors (i.e., epidemiologic transition) than have developing nations where not only is the transition occurring at a faster rate but is superimposed on an unresolved burden of infectious diseases. These risk behaviors exert long-term effects on national development and public health. Consider the following examples:

- Community participants in 18 nationwide focus groups in Zambia identified alcohol as the leading precipitating factor in the sexual transmission of HIV and in acquisition of other sexually transmitted diseases (personal communication, N. Luo, October 2004).
- A June 2005 consultation on the impact of alcohol on women, children, and families organized by the Centers for Disease Control and Prevention (CDC), other federal partners, and national and international stakeholders described many effects of alcohol that are detrimental to the health and economy of families in developing countries.
- In addition to its impact on women, children, and families, alcohol is implicated in over 60 other types of negative health outcomes, leading to 1.8 million deaths worldwide. Eighty percent of the excess mortality attributable to alcohol occurs in developing countries (2,8).
- The 2003 World Cancer Report identified tobacco and diet as the top two areas requiring intervention to reverse the rising global cancer rate, which is expected to reach 15 million in 2020 (14).
- All five behaviors discussed in this paper — tobacco use, alcohol consumption, overweight and obesity, low fruit and vegetable consumption, and physical inactivity — are associated with increased cardiovascular risk.

Some behavioral risk factors have implications for economic development. For instance, in Kenya, food production in tobacco-growing districts has decreased as farmers have shifted from food crops to tobacco to cater to increasing demands. More than half of the children in such areas were malnourished (15). The cost of purchasing tobacco and alcohol can consume 25% or more of a person's income in many developing countries (15). Such impact is likely to be increasingly concentrated among poor people in the poorer countries.

Despite 15 years of the global Safe Motherhood Initiative, overall maternal mortality remains unchanged at about 530,000 a year (16). As maternal and child health is directly linked to social, economic, and environmental conditions, many of the risks discussed here and their indirect implications for maternal health and family welfare have adverse effects on pregnancy and childhood development. Alcohol and tobacco are teratogens, and increases in exposure to teratogens place an added burden on the stagnant state of maternal and child health. Micronutrient malnutrition resulting from low fruit and vegetable intake can have adverse effects on the developing fetus (9,10).

Overweight and obesity contribute to other diseases. For example, type 2 diabetes mellitus, once a disease of older adults, now occurs in overweight and obese children and adolescents. Obesity early in life also has been associated with increased risk for certain cancers.

## Opportunities

Resource-constrained countries facing imminent public health challenges, such as AIDS or malaria, lack resources to either assess the impact of, or consider interventions for, modifiable behaviors for which the health impact is relatively long-term. For instance, despite the high prevalence of high-risk alcohol consumption among women, which ranges from 15% to 25% in countries of southern Africa (Demographic and Health Surveys, www.measuredhs.com [accessed January 12, 2006]), and despite reports of a correspondingly high incidence of fetal alcohol syndrome in those countries, legislative or public health measures to curb prenatal alcohol exposure are yet to be developed. Though it has been more than 25 years since Russell and colleagues demonstrated the effectiveness of brief clinician-delivered advice in persuading smokers to quit (17), such approaches are rarely employed in much of the developing world. Furthermore, when people have to deal with more imminent health threats and with competing life issues related to underdevelopment, it cannot be assumed that public information alone about health risks will lead to behavioral change.

Because the outcomes of most of the risk behaviors discussed here are long-term in comparison with categorical, single-vector infectious diseases, programmatic translation of interventions for these public health problems is challenging. However, many risk behaviors tend to cluster around the same population groups. Thus, bundled risk-reduction strategies could possibly be employed and may be appealing to policymakers, particularly in population groups in which adverse outcomes are mostly short-term. One such area where the impact of these modifiable behaviors is more immediate is pregnancy and fetal health. Women of childbearing age would benefit from a preconception or prenatal care approach that addresses some of the relevant modifiable behaviors.

WHO's focus on maternal and child health in 2005 provides a platform for sensitizing the public and policymakers to these asymmetrical behavioral threats to maternal and child health (15). Most efforts to improve pregnancy outcomes during the past 50 years have focused on promoting prenatal care and caring for pregnant women. In order to be effective, many interventions must be delivered before pregnancy and continued after delivery to detect, manage, modify, and control maternal behaviors, health conditions, and risk factors that contribute to adverse maternal and infant outcomes.

In recognition of this need, CDC recently convened a meeting of a select panel of stakeholders within government, academia, and nongovernmental organizations to develop recommendations for delivery of preconception health care services (18). This meeting also discussed programmatic experience in delivery of such services in China, Hong Kong, and South Korea. The consensus of the meeting was that efforts such as preconception care not only help address women's own health but also usefully complement the transfer of the concept of a healthy start from one generation to another. Participants also suggested that another opportunity lies in school-based health programs and that emphasis on a healthy start beginning before conception and continuing through adolescence would be a long-term investment that corresponds to the life-stage approach to health promotion.

Globalization is proceeding at a rapid pace, and lessons learned from it, such as the social marketing techniques employed in the consumption transition, should be used to facilitate best practices of public health in order to reach developing countries. Fast-track introduction of measures aimed at modifiable risk behaviors will require active governmental interventions to succeed. We know more than enough to act now. Lessons learned in developed countries from successful tobacco and alcohol control strategies would be useful in developing interventions in most developing countries. These include efforts aimed at taxation, advertising, counteradvertising, warning labels, and legislation. Bhutan's recent nationwide restriction on smoking is an example of how developing countries can embark on landmark public health policies (19). The 2002 Bellagio Conference on Nutrition Transition and many recent reports provide useful examples of nationally coordinated and systematic efforts in such diverse countries as Korea, Thailand, China, and Brazil aimed at physical inactivity, low fruit and vegetable intake, and obesity (9).

## The Way Forward

So far, global public health policy has been reactive; however, it needs to advance at the same or a faster rate than other economic sectors and in a complementary mode. Successfully globalized industries invest heavily in research and development to monitor market dynamics and adopt new strategies. Similarly, the health sector needs to invest in science and translational research capacity in developing countries to adopt, implement, and monitor interventions that are appropriate, replicable, and maintainable. Surveillance of both exposure and outcomes is foremost in increasing awareness and policy transitions. As emphasized in a recent editorial on China (20), well-structured, basic public health and applied public health science research needs to reach policymakers, not just public health practitioners. Because emerging health problems are similar in many countries, policies addressing behavioral risk factors in one emerging economy are likely to be applicable in others. However, action items may vary depending on the socioeconomic and political framework of each country or population subgroup. For instance, restricting access to tobacco for young people (policy) can be achieved through point-of-sales restrictions (action item) as in the United States or by reducing availability of tobacco products (action item) as in Bhutan.

Global public health will be only as good as that of the neediest country or region of the world. Public health programs alone cannot achieve the goal of reducing modifiable health risks. As reiterated in the 2005 Bangkok Charter for Health Promotion in a Globalized World (21), partnerships among global institutions devoted to public health, trade, finance, and environment are required to elevate public health dialogue and transform it into action. We are not short of examples of the success of such partnerships. Public health–automobile industry partnerships have played a crucial role in promoting safe driving behaviors, and public health–entertainment industry partnerships have embarked on many programs ranging from condom promotion to antismoking initiatives. Many countries allocate taxation from tobacco and alcohol for public health efforts. Adoption of such legislative measures and proven partnerships has been slow in developing countries, and greater momentum is needed.

Political stewardship emanating from global leaders such as that of the G8, an organization of leaders from the eight wealthiest nations, has helped elevate the response to emerging infectious disease, including development of various funding schemes (22). However, as stated in WHO's 2005 report, similar high-level political recognition of the looming chronic disease epidemic, its potential impact, and the needed response is yet to evolve (7). This report describes several interventions. Global debate and response can play a crucial role in policy transitions to address these and similar silent but significant threats to public health by making financial resources available both to develop the science base for interventions and to implement the interventions.

While the recognition and understanding by country leadership of the health issues that chronic diseases pose are important in effecting required policy changes, multilateral and bilateral funding mechanisms for health and social development should consider engaging resource-poor countries for change. The economic benefits of globalization can and should be directed to balance global health threats. Indeed, advancement of policy shifts and behavioral interventions would be more meaningful when implemented along with poverty-reduction programs and other structural interventions aimed at improving socioeconomic conditions that are the focus of the Millennium Development Goals developed by the United Nations (23). As we have learned from the tobacco epidemic, failing to take the right steps now can only further widen the development divide.

## Figures and Tables

**Table:** Table. Ranking of Selected Risk Behaviors as Leading Causes of Health Burden by Stage of Country Development, 2000

Risk Behavior	Rank		

	Developed Countries	Developing Countries	

		Low Mortality	High Mortality
Alcohol consumption	3	1	11
Tobacco use	1	3	9
Overweight and obesity	5	5	14
Physical inactivity	7	14	13
Low fruit and vegetable intake	6	7	15

Source: World Health Organization (2).
